# Analysis of Predictors for Lymph Node Metastasis in Patients with Superficial Esophageal Carcinoma

**DOI:** 10.1155/2016/3797615

**Published:** 2016-10-05

**Authors:** Ruzhen Jia, Qinsong Luan, Jing Wang, Dongsheng Hou, Shulei Zhao

**Affiliations:** ^1^Department of Gastroenterology, Shandong Provincial Hospital Affiliated to Shandong University, 324 Jingwu Weiqi Rd, Jinan 250021, China; ^2^Department of Gastroenterology, Qilu Hospital of Shandong University, 324 Jingwu Weiqi Rd, Jinan 250021, China

## Abstract

In order to predict related risk factors for lymph node metastasis (LNM) in patients with superficial esophageal carcinoma (SEC) and provide reference for endoscopic minimally invasive treatment, we included a total of 93 patients with superficial esophageal carcinoma who have underwent esophagectomy and lymph node dissection from 2010 to 2015. The depth of invasion was remeasured and classified into 6 groups according to their wall penetration. The prediction model was founded based on the independent risk factors. The results shows that lymph node metastasis of m1, m2, m3, sm1, sm2, and sm3 of superficial esophageal carcinoma was 0%, 0%, 5.3%, 8.7%, 17.6%, and 37.5%, respectively. The tumor size, differentiation, and lymphvascular invasion were also significantly related to lymph node metastasis by univariate analysis. Multivariate analysis showed that the depth of invasion and lymphovascular invasion were independent risk factors of lymph node metastasis. A prediction model for lymph node metastasis was established as follows: *p* = *e* ^*x*^/(1 + *e* ^*x*^), and *x* = −5.469 + 0.839 × depth of invasion + 1.992 × lymphavascular metastasis. The area under ROC curve was 0.858 (95% CI: 0.757–0.959). It was also shown that the depth of invasion was related to tumor differentiation, macroscopic type, and tumor size.

## 1. Introduction

Superficial esophageal carcinoma (SEC) includes mucosal and submucosal carcinomas with or without the presence of lymph node metastasis. The prognosis is good after surgery. Esophagectomy with lymph node dissection remains standard therapy for SEC. A variety of complications could develop after surgery [1–3], and the quality of patient's life could be greatly influenced because of the modification of esophagus [4, 5].

Endoscopic therapy is indicated for the management of superficial esophageal carcinoma (SEC) that has a minimal risk of lymph node metastasis. To those without lymph node metastasis, endoscopic resection has been suggested as alternative to esophagectomy in the treatment of these lesions; the effect can be matched with surgery [6].

Endoscopic resection can preserve the whole esophagus and does not influence the quality of life. On the other hand, esophagectomy with lymph node dissection should be applied for SEC with high risks for lymph node metastasis. Therefore, the occurrence of lymph node metastasis in superficial esophageal carcinoma is important for the selection of treatment options. In clinical practice, it is very difficult to determine lymph node metastasis in SEC [7].

In order to find risk factors related to lymph node metastasis and provide clinical guidance for endoscopic treatment in SEC, we conducted a retrospective study to identify clinicopathologic predictors for lymph node metastasis in SEC. The aim of this study was to select reliable patients without lymph node metastasis.

## 2. Patients and Methods

### 2.1. Patients

Between 2010 and 2015, 1905 patients were treated with surgery for esophageal cancer in Shandong Provincial Hospital Affiliated to Shandong University. Of those patients, 93 showed SEC, including 37 mucosal lesions and 56 submucosal lesions. These were 72 men and 21 women with a median age of 58 (range: 44–78) years.

### 2.2. Methods

All patients underwent esophagectomy and lymph node dissection. The surgical procedures were performed by thoracoscopic or open cardiac surgery depending on the surgeon's preferences and the patient's surgical history. The specimens were removed en bloc and the lymph nodes from the specimens were dissected according to a standardized protocol. Lymph node metastasis was found in 12 of the 93 patients (12.9%), including 1 intramucosal carcinoma (1.1%) and 11 submucosal carcinomas (11.8%).

The lesions were divided into 3 equal layers of the mucosa (m1, m2, and m3) and submucosa (sm1, sm2, and sm3) according to Japanese classification of the esophageal carcinoma [8]. The depth of infiltration was measured at the deepest point of penetration of the cancer cells in the corresponding layer. All patients were categorized into three groups according to the macroscopic type: type 0-I (protruded type), type 0-II (flat type), and type 0-III (excavated type). D2-40 immunostaining was used as a method to assess the lymphovascular invasion. The lymphovascular infiltration at the submucosal area of the cancer was classified as either negative or positive. Tumor differentiation was determined in accordance with the WHO criteria and subdivided into three groups: well, moderately, and poorly differentiated. Possible risk factors, such as tumor location, pathologic type, and tumor size, were also included for the analysis of lymph node metastasis. Two pathologists performed histopathological review in each patient; disagreements were resolved by consultation with a third investigator.

### 2.3. Statistical Analysis

Contingency tables were analyzed using Fisher's exact test. The relevance of increasing frequency of lymph node metastasis was analyzed using Chi-square trend test. With the logistic regression test, we identified the independent risk factors of lymph metastasis. Spearman nonparameter method was performed to analyze the correlation between categorical variables. All statistical analyses were analyzed with the Statistical Package for the Social Sciences (SPSS) for Windows, Version 19.0 (SPSS Inc., Chicago, IL).* P* value < 0.05 was considered to be statistically significant.

## 3. Results

The relationships between sex, age, tumor location, tumor size, tumor differentiation, macroscopic type, depth of invasion, lymphovascular infiltration, and lymph node metastasis were shown in Table 1. Lymph node metastasis was significantly associated with deeper tumor layer, larger tumor size, undifferentiated pathological type, and lymphovascular infiltration. The rate of lymph node metastasis according to three-third of mucosal (m1, m2, and m3) or submucosal infiltration (sm1, sm2, and sm3) was 0%, 0%, 5.3%, 8.7%, 17.6%, and 37.5%, respectively. Different macroscopic types trended toward association with lymph node metastasis (*P* = 0.064). Patients with 0-II type had the lowest (4.9%) rate of lymph node metastasis whereas 0-I type had the highest rate (20.9%). The rate of lymph node metastasis between different depths of invasion was significantly different. There were no significant differences between lymph node metastasis and sex, age, and tumor location.

**Figure 1:**
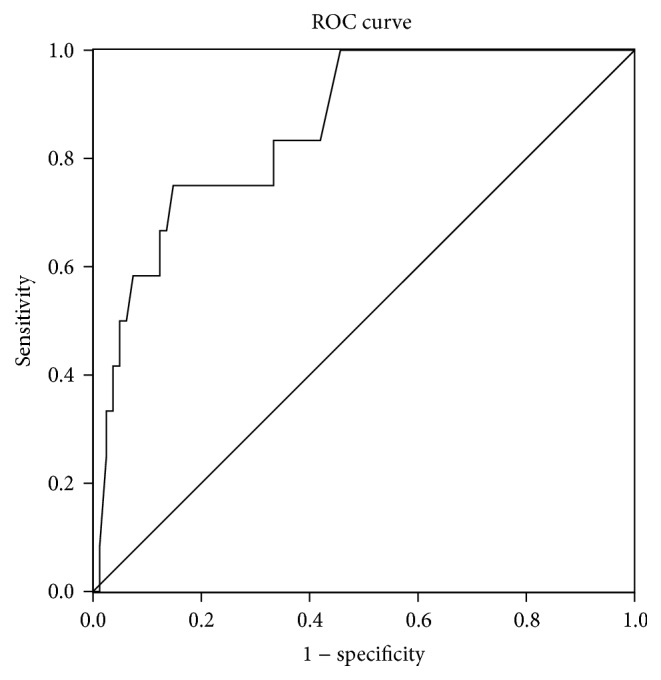
ROC curve for the prediction model. Diagonal segments are produced by ties.

All variables significant in univariate analysis were entered for multivariate analysis with binary logistic regression. The results showed that depth of invasion and lymphovascular infiltration were independent risk factors for lymph node metastasis (Table 2). A formula was developed to estimate the probability of having lymph node metastasis on the basis of the results of the binary logistic regression analysis (Figure 1). A score is calculated using depth of invasion and lymphovascular infiltration: *p* = *e* ^*x*^/(1 + *e* ^*x*^), *x* = −5.469 + 0.839 × depth of invasion + 1.992 × lymphovascular metastasis. Depth of invasion was encoded as 1, 2, 3, 4, 5, and 6. Tumor with or without lymphovascular infiltration was encoded as 0 and 1, respectively. The area under the receiver operating characteristic (ROC) curve was 0.858 (95% CI: 0.757–0.959).

We identified risk factors for depth of tumor invasion by Spearman correlational analysis (Table 3). It demonstrated that tumor differentiation, tumor size, and macroscopic type were risk factors for tumor invasion (*r* = 0.0656, *P* < 0.0001; *r* = 0.475, *P* < 0.0001; *r* = −0.494, *P* < 0.0001).

## 4. Discussion

With the development of endoscopic technology and the promotion of early screening of esophageal carcinoma, the detection rate of superficial esophageal carcinoma increased year by year. Superficial esophageal carcinoma patients without lymph node metastasis can be cured by endoscopic resection, such as endoscopic submucosal dissection (ESD) or endoscopic mucosal resection (EMR) [9].

Kato et al. [10] reported 92 patients treated with esophagectomy for superficial esophageal carcinoma. They found that cancer invasion was limited to the mucosa in 24 cases and only 1 patient (4.2%) had lymph node involvement, whereas 35.3% patients of the submucosal invasion group had lymph node metastases.

When patients had lymph node metastasis, endoscopic resection cannot achieve the purpose of radical cure. Lymph node metastasis is an important biological characteristic of esophageal carcinoma, which can affect the treatment of esophageal cancer. In Japan and some Western countries, previous studies of patients with SEC showed that 1% to 11% of mucosal carcinomas showed lymph node metastasis, whereas the incidence of lymph node metastasis in submucosal carcinomas was 15.6% to 49% [11–15]. Eguchi et al. [16] investigated the association between histopathological factors and LNM in 464 consecutive patients with superficial squamous cell carcinoma of the esophagus who had undergone a radical esophagectomy with lymph node dissection. LNM was found in 0, 5.6, 18.0, 53.1, and 53.9% of the m1, m2, m3, sm1, and sm2/3 lesions. In China, Li et al. [17] clarified the pattern of lymphatic spread in patients with superficial esophageal squamous cell carcinoma and analyze the factors potentially related to LNMs. LNMs were reported in 0%, 0%, 11.8%, 24.0%, 20.5%, and 43.8% of the m1, m2, m3, sm1, sm2, and sm3 carcinoma, respectively.

Patients included in the study were divided into 6 groups according to the depth of infiltration: mucosal tumors were classified as m1, m2, and m3 and submucosal tumors as sm1, sm2, and sm3. In our series, mucosal carcinomas of the m1 and m2 layer showed no lymph node metastasis. The lymph node metastasis springs from 0% to 5.3% when the tumor infiltrated the m3 layer. When carcinoma infiltrated the submucosa, the probability of lymph node metastasis increased from 8.7% in the sm1 layer to 37.5% in the sm3 layer. We concluded that m1 infiltration and m2 infiltration are the absolute indications of endoscopic treatment and m3 infiltration and sm1 infiltration are the relative indications, but they need to be followed up closely; surgical treatment is needed when the infiltration reaches sm2 or sm3.

Through the study of these 93 cases of superficial esophageal cancer, there was a significant correlation between the degree of differentiation and lymph node metastasis. The lower the degree of differentiation, the higher the rate of lymph node metastasis. In the study, 33 cases of high differentiated esophageal carcinoma had no lymph node metastasis, while the rates of lymph node metastasis in middle and low differentiation group were 16.7% and 27.8%, respectively. We concluded that well-differentiated tumor is the indication of endoscopic treatment.

The size of the tumor is helpful to the prediction of lymph node metastasis of superficial esophageal carcinoma. The lymph node metastasis rate of tumor diameter in <1 cm, 1-2 cm, 2-3 cm, and >3 cm was 0%, 17.1%, 15%, and 33.3%, respectively. When the tumor diameter exceeds 3 cm, all infiltrated in submucosa (9/9), and the lymph node metastasis rate is high, it is not suitable for endoscopic treatment. No case of superficial esophageal carcinoma with a diameter of less than 1 cm had lymph node metastasis (0/26); it is an indication of endoscopic resection.

We studied the relationship between the macroscopic type of SEC and lymph node metastasis. The results showed that the incidence of lymph node metastasis was 20.9% of 0-I type, 11.1% of 0-III type, and 4.9% of the 0-II type. The risk of the lymph node metastasis of 0-I and 0-III type was higher than 0-II type, so we recommend that the depth of infiltration can be further evaluated by magnifying endoscopy and endoscopic ultrasonography [18, 19].

Our study also showed that the risk of lymph node metastasis in SEC patients with lymphovascular invasion was significantly higher than that in patients with no lymphovascular invasion (32.1% versus 4.6%). Therefore, if the specimen appears to have lymphovascular infiltration after endoscopic resection, the patient has a high probability of lymph node metastasis. We recommend close follow-up or additional surgical treatment.

Univariate analysis showed that tumor size, differentiation, depth of invasion, and lymphovascular metastasis were correlated with lymph node metastasis. It was found that only depth of invasion and lymphovascular infiltration were independent predictors of lymph node metastasis by logistic regression analysis. A prediction model for lymph node metastasis was established according to the two independent predictors; it has high accuracy, which has certain guiding significance for clinical work. Compared with the previously published 6 kinds of prediction models [20–25], the AUC value of our model is higher than most of them. However, there are only two factors included in our model, with less interference factors, showing good sensitivity and specificity.

In addition, we have studied the risk factors for the depth of the invasion and lymphovascular infiltration in SEC patients. We found that there was a significant correlation between differentiation, tumor size, macroscopic type, and depth of invasion. Tumor with low differentiation, larger diameter, and nonflat macroscopic type were easy to infiltrate the submucosa.

This study has some drawbacks: firstly, it is a retrospective study; secondly, the sample size is small, and, after further grouping, the number of cases per group was less. Further prospective studies with larger sample sizes are needed to verify the results.

In conclusion, the depth of invasion, tumor differentiation, tumor size, and lymphovascular infiltration were closely associated with lymph node metastasis, and the depths of invasion and lymphovascular invasion were independent risk factors of lymph node metastasis in superficial esophageal carcinoma.

## Figures and Tables

**Table 1:** Relationship between clinicopathological factors and lymph node metastasis.

Correlational analyses		Cases	Cases of lymph node metastasis	Rate of lymph node metastasis	*P* value
Sex	Male	72	10	13.9	0.728
	Female	21	2	9.5	

Age	≤60 years	60	9	15.0	0.529
	>60 years	33	3	9.1	

Tumor size	≤10 mm	26	0	0.0	0.017
	11–20 mm	35	6	17.1	
	21–30 mm	20	3	15.0	
	≥31 mm	9	3	33.3	

Pathological type	Squamous carcinoma	86	12	14.0	0.589
	Others	7	0	0.0	

Differentiation	Good	33	0	0.0	0.005
	Moderate	42	7	16.7	
	Low	18	5	27.8	

Macroscopic type	0-I	43	9	20.9	0.064
	0-II	41	2	4.9	
	0-III	9	1	11.1	

Location	Upper	3	0	0.0	0.578
	Middle	56	6	10.7	
	Low	34	6	17.6	

Depth of invasion	m1	9	0	0.0	0.046
	m2	9	0	0.0	
	m3	19	1	5.3	
	sm1	23	2	8.7	
	sm2	17	3	17.6	
	sm3	16	6	37.5	

Lymphvacular infiltration	Negative	65	3	4.6	0.001
	Positive	28	9	32.1	

*P* values were calculated by the Fisher exact probability test.

**Table 2:** Multivariate analysis of risk factors for lymph node metastasis.

Variable	*B*	*P*	OR (95% CI)
Differentiation	0.140	0.822	1.151 (0.338–3.915)
Tumor size	−0.384	0.476	0.681 (0.237–1.958)
Macroscopic type	−0.434	0.503	0.648 (0.182–2.309)
Depth of invasion	0.839	0.048	2.313 (1.006–5.319)
Lymphvascular infiltration	1.992	0.038	7.330 (1.117–48.083)
Constant	−5.469	0.016	0.004

**Table 3:** Relationship between clinicopathologic factors and depth of invasion.

Variable		Total	m	sm	sm (%)	*P*
Differentiation	Good	33	26	7	21	<0.001
	Moderate	42	11	31	74	
	Low	18	0	18	100	

Tumor size	≤10 mm	26	14	12	50	0.03
	11–20 mm	35	13	22	60	
	21–30 mm	20	7	13	70	
	≥31 mm	9	0	9	100	

Macroscopic type	0-I + 0-III	52	10	42	80	<0.001
	0-II	41	27	14	30	
